# Ru Single Atoms on One-Dimensional CF@g-C_3_N_4_ Hierarchy as Highly Stable Catalysts for Aqueous Levulinic Acid Hydrogenation

**DOI:** 10.3390/ma15217464

**Published:** 2022-10-25

**Authors:** Ying Yang, Suoying Zhang, Lin Gu, Shijie Hao

**Affiliations:** 1 State Key Laboratory of Heavy Oil Processing, China University of Petroleum, Changping, Beijing 102249, China; 2 Institution of Advanced Materials, Nanjing Tech University, Nanjing 211816, China

**Keywords:** Ru single atom, one-dimensional hierarchy, levulinic acid, γ-valerolactone, stability

## Abstract

Herein, we report a stable catalyst with Ru single atoms anchored on a one-dimensional carbon fiber@graphitic carbon nitride hierarchy, by assembling wet wipes composed of fiber-derived carbon fiber (CF), melamine-derived graphitic carbon nitride (g-C3N4) and RuCl3 before NaBH4 reduction. The atomically dispersed Ru species (3.0 wt%) are tightly attached via N-coordination provided by exterior g-C3N4 nanosheets, and further stabilized by the interior mesoporous CF. The obtained CF@g-C3N4–Ru SAs catalyst can be cycled six times without notable leaching of Ru or loss of GVL yield in the acidic media. This catalyst is more stable than Ru nanoparticles supported on CF@g-C3N4, as well as Ru single atoms anchored on CF and g-C3N4, and proves to be one of the most efficient metal catalysts for aqueous LA hydrogenation to γ-valerolactone (GVL). The isolated Ru atoms by strong N-coordination, and their enhanced electron/mass transfer afforded by the one-dimensional hierarchy, can be responsible for the excellent durability of CF@g-C3N4–Ru SAs under harsh reaction conditions.

## 1. Introduction

The gradual consumption of fossil resources and the growing attention directed toward global climate change have stimulated the efforts for the sustainable production of materials, chemicals and fuels [1]. The hydrogenation of cellulosic biomass-derived levulinic acid (LA) into γ-valerolactone (GVL) is a key reaction included in many biorefinery schemes for the production of renewable chemicals and fuels [2]. GVL is not only a key and safe intermediate for fine chemical and liquid fuel synthesis, but also a building block for polymers, a solvent and a flavoring agent [3]. However, achieving a high yield of GVL repeatedly is very difficult, since upgrading cellulosic LA is usually operated in aqueous media at a high temperature (>130 °C). Besides the impurities (such as sulfuric acid and formic acid) in LA feeds, abundant H^+^ produced by the dissociation of LA and the ionization of subcritical water from the solvent/co-product makes the reaction media very acidic (pH ≈ 1), as the conversion of LA proceeds at a high temperature [4]. The reaction medium is normally oxygen functionalized, polar and even corrosive. In this case, the catalysts are easily deactivated owing to metal sintering, leaching, support collapse/destruction as well as the formation of carbonaceous deposits and process-derived impurities to poison active sites [5]. Such a tough reaction environment raises a significant challenge for the durability of the metal catalysts. Ru-based catalysts prove to be more persistent in acidic media as compared to non-precious metal sites. However, the durability of Ru catalysts under aqueous and acidic reaction conditions remains a concern to be addressed. It has been demonstrated that the supported Ru species are thermodynamically unstable and tend to sinter into larger particles in the presence of water, even at room temperature [6]. Additionally, these Ru sites can be poisoned in the presence of acids and oxidants, also leading to the deactivation of the Ru catalysts as they are involved in the aqueous LA hydrogenation [7,8]. In this regard, it is highly desirable to construct persistent Ru sites that are active under repeated reactions, even exposed to water, acid and/or oxidant.

With the emergence of the concept of single-atom catalysis, it is anticipated that stable Ru catalysts will be developed by constructing atomically dispersed Ru sites on a certain supporting matrix via a strong metal–support interaction (SMSI). These Ru atoms can be isolated by the low Z elements surrounding, and the concerned aggregation, leaching, poisoning and oxidation would be alleviated toward aqueous LA hydrogenation [9]. Several Ru single-atom catalysts (SACs) have been fabricated by confinement of Ru single atoms into carbon nitride [10,11], N-doped graphene [12] and a metal–organic framework (such as ZIF, UiO-66, etc.)-derived carbons [13,14,15], showing excellent stability in catalytic reactions such as electrocatalytic H_2_/O_2_ evolution, O_2_ reduction and quinoline/vanillin hydrogenation. The construction of single-atom sites to enhance catalytic stability has been recently demonstrated in biomass valorization. It was found that the Ni-N-C SAC delivered excellent stability towards the direct conversion of cellulose into ethylene glycol in water, and no metal aggregation or leaching was detected after seven consecutive runs [16]. Another SAC with Co atoms incorporated into the sulfur vacancies of MoS_2_ monolayer sheets displays enhanced stability compared to traditional CoMoS_2_ for the hydrodeoxygenation of 4-methylphenol into toluene. Deactivation cannot be found for this Co SAC, even when cycled seven times [17]. Nevertheless, highly stable metal SACs for LA hydrogenation under harsh reaction conditions are scarcely reported. To the best of our knowledge, only one stable SAC for aqueous LA hydrogenation was reported by Zhang and co-workers. The Ru single atoms (0.85 wt%) were dispersed on nanotetragonal ZrO_2_ and embedded in the amorphous carbon, which was achieved by the impregnation of RuCl_3_ into UiO-66 derived ZrO_2_@C support [14]. This catalyst exhibits excellent stability with no apparent drop in catalytic performance observed in six consecutive runs in water and three consecutive runs in acidic media (pH ≈ 1). Great progress has been made to develop stable Ru catalysts by constructing Ru single-atom sites. However, few investigations give importance to the supporting matrix and its synergism in single atoms to enhance the stability of catalysts under harsh reaction conditions. The low porosity of metal oxide supports and their vulnerability under acidic/hydrothermal conditions deactivate Ru single atoms. This calls for a persistent supporting matrix that not only stabilizes Ru single atoms, but also provides mesoporous arrays to facilitate mass transfer to extend catalyst life. Mesoporous carbons are chemically inert and stable, but the absence of coordination sites may lead to the leaching of loosely attached metal species during reactions. Graphic C_3_N_4_ can stabilize single atoms by N-coordination, but the sheet-like g-C_3_N_4_ is less porous and tends to aggregate during repeated reactions. An ideal supporting matrix for a stable Ru SAC can not only regulate the geometric and electronic structure of single-metal sites based on SMSI, but also provide persistent porosity and inertness under harsh conditions to guarantee the durability of the entire catalyst. However, for aqueous LA hydrogenation, the rational design of an inert support with well-defined coordination sites and a mesoporous structure to construct stable Ru SACs has never been achieved.

Herein, we report a stable Ru catalyst with atomically dispersed Ru species (3.0 wt%) anchored on a one-dimensional CF@g-C_3_N_4_ hierarchy. What deserves to be mentioned is the construction of the core–shell structured CF@g-C_3_N_4_ supporting matrix. Such a one-dimensional hierarchy provides precise N-coordination sites to stabilize/regulate Ru single atoms, by the dispersion of exterior g-C_3_N_4_ nanosheets. Additionally, the mesoporous structure of the CF@g-C_3_N_4_ hierarchy accelerates the electron/mass transfer and survives in repeated reactions under harsh conditions. As expected, the obtained CF@g-C_3_N_4_–Ru SAs exhibits outstanding durability towards aqueous LA hydrogenation in acidic media.

## 2. Experimental

### 2.1. Chemicals

All of the chemicals were commercially obtained and were used without further purification. Ruthenium trichloride (RuCl_3_·xH_2_O, 38 wt%) was purchased from Aladdin Industrial Corporation, Shanghai. Melamine (AR, 99%) and sodium borohydride (NaBH4, 99%) were purchased from Tianjin Guangfu Technology Development Co. Ltd. Nitric acid (HNO_3_, 68 wt%), hydrochloric acid (HCl, 36 wt%), ethanol (EtOH, 95%) and acetone (AR, 99%) were purchased from Beijing Chemical Works. The wet wipes fiber was purchased from the market, produced by PHILIPS and containing a small quantity of titanium (0.27 wt%).

### 2.2. Sample Preparation

The wet wipes fiber (WWF, 2 cm × 2 cm) obtained for daily wipe were cleaned by acetone, hydrochloric acid (0.1 M), ethanol and deionized water under ultrasonic treatment in turn, and the obtained WWF was immersed into the HNO3 aqueous solution (2.0 M) at 60 °C for 1 h to ensure a clean surface. The dried WWF was then pyrolyzed at 500 °C for 2 h in N2 atmosphere, yielding the carbon fiber, CF (40% yield). The melamine was calcinated at 550 °C in a furnace for 3 h, affording graphitic carbon nitride, g-C_3_N_4_ (65% yield, 99% purity). Subsequently, 0.1 g of g-C_3_N_4_ combined with 0.1 g of CF was dispersed in 1000 mL of water using sonication for 30 min. Then, 20 mg of RuCl_3_·xH_2_O was introduced and the obtained mixture was stirred for 12 h, followed by addition of 0.5 g NaBH_4_. The resulting mixture was further stirred for 6 h to produce CF@g-C_3_N_4_–Ru SAs catalyst. The synthesis of CF@g-C_3_N_4_–Ru NPs was performed according to the procedure mentioned above with slight modification. Typically, 0.1 g of g-C_3_N_4_ combined with 0.1 g of CF was dispersed in 1000 mL of water using sonication for 30 min, followed by introduction of 20 mg of RuCl_3_, and the resulting mixture was stirred for 12 h. The Ru^3+^-impregnated CF@g-C_3_N_4_ was obtained and then pyrolyzed at 500 °C under N_2_ atmosphere for 1 h, yielding CF@g-C_3_N_4_–Ru NPs. The synthesis of g-C_3_N_4_–Ru and CF–Ru was also performed as the procedure mentioned for CF@g-C_3_N_4_–Ru SAs synthesis, but without CF or g-C_3_N_4_ involved.

### 2.3. Characterization

Powder X-ray diffraction patterns of samples were obtained on a Rigaku D/max 2500Pc X-ray powder diffractometer using a Cu Kα radiation (λ = 0.15418 nm). TEM images were recorded on a Hitachi-7700 worked at 100 kV. High-resolution TEM images were obtained by a FEI Tecnai G2 F20 S-Twin HRTEM working at 200 kV. Atomic resolution HAADF-STEM images were obtained by using a Titan 80–300 scanning/transmission electron microscope operator at 300 kV, equipped with a probe spherical aberration corrector. Surface area analysis was performed on a JW-BK100 static volumetric gas adsorption instrument and the surface area was calculated by using the Brunauer–Emmett–Teller (BET) method. X-ray photoelectron spectroscopy (XPS) was performed on a Thermo Scientific Escalab 250Xi X-ray photoelectron spectrometer. The Ru content was estimated by ICP-OES analysis conducted on a Thermo Icap 6300 emission spectrometer. Before the test, a certain amount of vacuum-dried sample was placed in a microwave digestion tank, and dissolved in 4 mL of aqua fortis solution mixed with 1 mL hydrogen peroxide. Microwave digestion was carried out for 30 min to completely dissolve the metal species. After cooling, each solution was filtered through a 0.45 μm poly-ethersulfone filter. X-ray absorption fine structure (XAFS) was carried out on the 20-BM-B beamline of Advanced Photo Source (APS) at Argonne National Laboratory. The XAFS data were obtained in the transmission mode at the Ru K-edge (22,117.0 eV). The XAFS data were processed using the Athena software for background removal, post-edge normalization and X-ray absorption near edge structure (XANES) analysis. The extended X-ray absorption fine structure (EXAFS) was analyzed using Artemis software, which implemented FEFF. The EXAFS data reduction was conducted by utilizing the standard procedures. The EXAFS function, χ, was obtained by subtracting the post-edge background from the overall absorption and then normalized with respect to the edge jump step. Subsequently, k^2^-weighted χ(k) data in k space were Fourier transformed to r space in order to separate the EXAFS contributions from the different coordination shells.

### 2.4. Catalytic Test

The LA hydrogenation reactions were carried out in a magnetically stirred Parr 4560 autoclave. In a typical procedure, 30 mg of catalyst, 40 mL of H_2_O and 0.12 g of LA were introduced into the autoclave. The sealed autoclave was charged and deflated with N2 three times before it was pressurized with H2 to 4.5 MPa at room temperature and heated at 150 °C for 2 h. The separated liquid solution was quantitatively analyzed with gas chromatography (SP-6890) using 1, 4-dioxane as the internal standard.

## 3. Results and Discussion

**Scheme 1:**
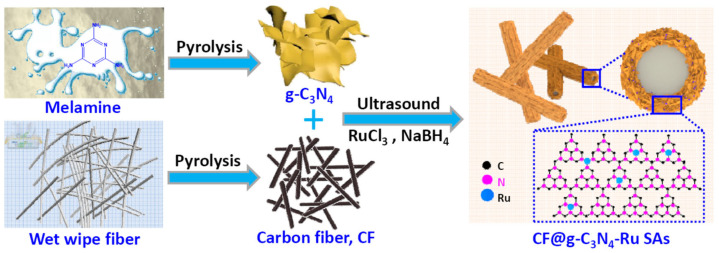
Schematic illustration of the synthetic process for CF@g-C_3_N_4_–Ru SAs.

The schematic description of CF@g-C_3_N_4_–Ru SAs synthesis is shown in Scheme 1. As illustrated, the wet wipes fiber (WWF) and melamine were utilized as precursors and pyrolyzed in N_2_ atmosphere to produce carbon fiber (CF) and graphitic carbon nitride (g-C_3_N_4_), respectively. The ultrasonic treatment of g-C_3_N_4_ and CF in water not only exfoliates g-C_3_N_4_ in favor of capturing more Ru^3+^ in the subsequent impregnation, but also facilitates the formation of a core–shell structured CF@g-C_3_N_4_ hierarchy. Considering the aggregation tendency of Ru species at a high temperature, the reduction in Ru^3+^-impregnated CF@g-C_3_N_4_ was performed using NaBH_4_ at room temperature to synthesize CF@g-C_3_N_4_–Ru SAs. Otherwise, the nanoparticle catalyst, denoted as CF@g-C_3_N_4_–Ru NPs, was formed by heating of the Ru^3+^-coordinated CF@g-C_3_N_4_ intermediate at 500 °C in an N_2_ flow. Atomic Ru species anchored on a single CF and g-C_3_N_4_ support, denoted as CF–Ru and g-C_3_N_4_–Ru, respectively, were also prepared for comparison (please refer to the supporting information).

**Figure 1:**
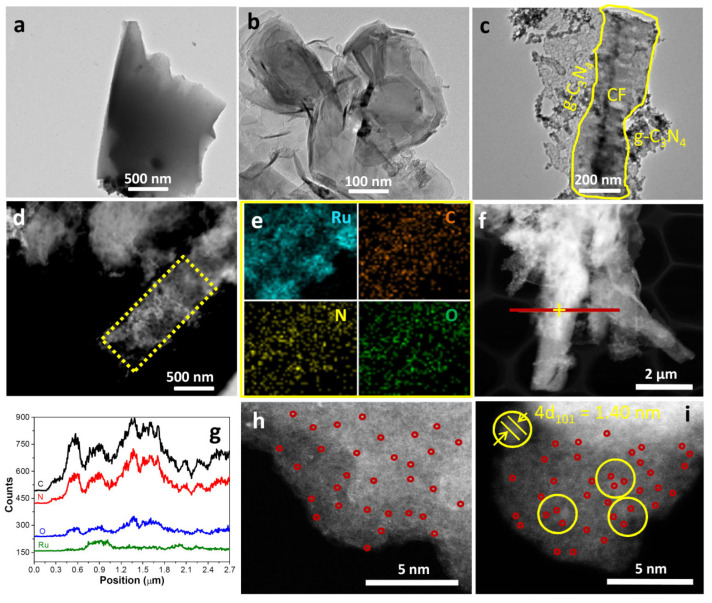
TEM images of (**a**) CF, (**b**) g-C_3_N_4_ and (**c**) CF@g-C_3_N_4_–Ru SAs, (**d**,**f**) HAADF-STEM images and (**e**,**g**) the corresponding elemental maps and line scanning profiles of CF@g-C_3_N_4_–Ru SAs, and (**h**,**i**) aberration-corrected STEM image of CF@g-C_3_N_4_–Ru SAs.

The WWF-derived CF is very smooth and has an average diameter of ca. 2 μm based on the transmission electron microscopy (TEM) observation (Figure 1a). g-C_3_N_4_ originated from melamine contains thin layers as shown in Figure 1b, which were then coated on the surface of CF and followed by RuCl_3_ impregnation and NaBH_4_ reduction. The resulting CF@g-C_3_N_4_–Ru SAs has a core–shell configuration with g-C_3_N_4_ nanosheets around the fibrous core, and no obvious Ru clusters or nanoparticles can be observed (Figure 1c), whereas the existence of homogeneously distributed Ru species can be revealed by the high-angle annular dark field scanning TEM (HAADF-STEM) image and elemental maps (Figure 1d,e). The HAADF-STEM image along with the line scanning profile across a CF@g-C_3_N_4_–Ru SAs fiber shows that both the N and Ru elements are located on the surface, while carbon is distributed inside (Figure 1f,g). These results further demonstrate the core–shell configuration and preliminarily indicate the well-dispersed Ru species on the exterior g-C_3_N_4_. In order to reveal the Ru structure, an observation was performed by aberration-corrected scanning TEM (AC-STEM). As shown in Figure 1h, bright dots with atomic resolution can be detected and are homogeneously distributed. Due to the remarkable difference in Z-contrast between Ru and N/C, these bright dots can be determined to be atomically dispersed Ru species, indicating the formation of Ru single atoms. We also observed some Ru atoms that are embedded into the (101) plane of TiO_2_ (Figure 1i), marked by the yellow circles. In this study, CF@g-C_3_N_4_–Ru NPs was also examined, and it displays a core–shell configuration decorated with numerous Ru nanoparticles showing an average diameter of 10.1 nm (Figure S1). This strongly suggests the critical role of NaBH_4_ reduction in the synthesis of Ru SACs. The Ru mass loading for CF@g-C_3_N_4_–Ru SAs is determined to be 3.0% by ICP-OES analysis (Table S1), which is twice more than that for CF@g-C_3_N_4_–Ru NPs (1.3 wt%), and lower than that for CF–Ru (4.1 wt%) and g-C_3_N_4_–Ru (4.5 wt%).

**Figure 2:**
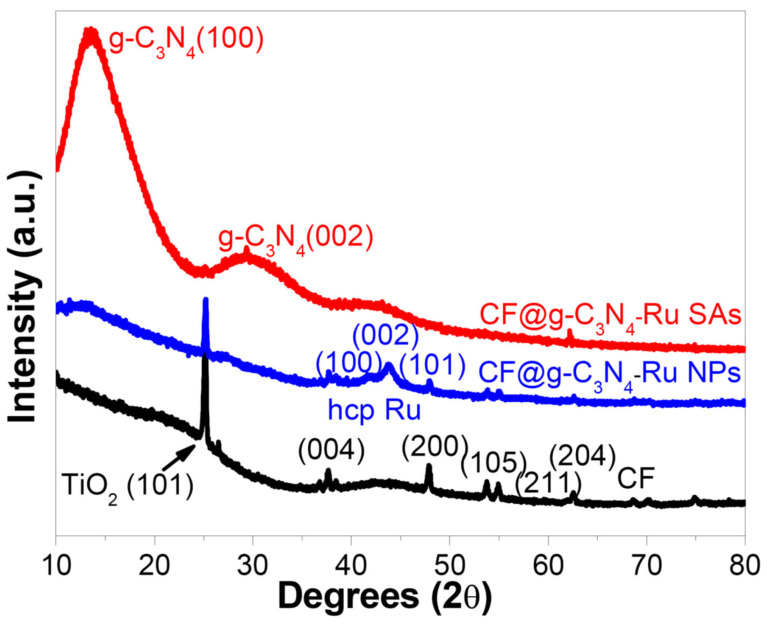
XRD patterns of different samples.

The crystalline structure of different samples was investigated by X-ray diffraction (XRD). As shown in Figure 2, the XRD pattern of CF@g-C_3_N_4_–Ru SAs shows two obvious diffraction peaks at 13.4° and 27.5°, which can be indexed to the (100) and (002) planes of g-C_3_N_4_ (JCPDS No.: 87-1562), respectively, and another weak diffraction at 43.3° which can be ascribed to the (002) facet of the hexagonal carbon [18,19]. Characteristic diffractions corresponding to crystalline Ru cannot be detected, in accordance with the atomically dispersed Ru species observed by AC-STEM. Comparatively, the XRD pattern of CF@g-C_3_N_4_–Ru NPs provides broad but clear (100), (002) and (101) diffractions that can be ascribed to *hcp*-structured Ru NPs (JCPDS No.: 06-0663) [20]. The particle size is determined to be 9.8 nm, calculated using the Scherer equation, and confirming the TEM observation. A sharp diffraction at 25.2° combined with several weak peaks at 37.8°, 47.8°, 53.7°, 55.1° and 62.6° is also detected as those for CF, referring to the (101), (004), (200), (105), (211) and (204) lattice planes of TiO_2_ (JCPDS No.: 71-1167), respectively [21]. This is because WWF contains 0.27 wt% of Ti, which was transformed into TiO_2_ after pyrolysis. It is noteworthy that as the activation/reduction was performed a high temperature in N_2_ flow, TiO_2_ was retained for CF@g-C_3_N_4_–Ru NPs. Otherwise, it was mostly removed for CF@g-C_3_N_4_–Ru SAs as it was reduced by NaBH_4_. We also noticed that two diffraction peaks assigned to g-C_3_N_4_ are vague for CF@g-C_3_N_4_–Ru NPs, which likely indicates that the secondary pyrolysis destroyed the crystalline structure of g-C_3_N_4_. It is clear that heat treatment at 500 °C not only accelerates the mobility of the reduced Ru species, but also provides another pathway to accelerate the aggregation to Ru NPs by the release of N-coordinated Ru species. By contrast, NaBH_4_ reduction is not only beneficial to the synthesis of Ru SAs, but also helpful to remove the TiO_2_ impurities.

**Figure 3:**
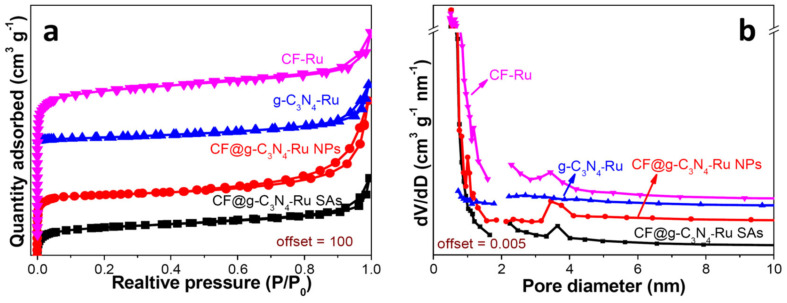
(**a**) N_2_ adsorption/desorption isotherm and (**b**) the corresponding pore size distribution curve for different Ru-containing catalysts.

N_2_ adsorption/desorption reveals a mixed I and IV type isotherm with a distinct H_4_ type hysteresis loop for CF@g-C_3_N_4_–Ru SAs (Figure 3a), indicative of hierarchical micro- and mesoporous materials with slit pores [22]. The mesopore size shows a mono distribution centered at ca. 3.7 nm, while the micropore size exhibits a bimodal distribution centered at 0.52 and 0.60 nm (Figure 3b). These hierarchical pores originate from the core–shell structured CF@g-C_3_N_4_ supporting matrix, which may facilitate the reactant/product transportation during the aqueous-phase reactions. Moreover, CF@g-C_3_N_4_–Ru SAs shows a BET surface area of 380 m^2^ g^−1^ and a total pore volume of 0.57 cm^3^ g^−1^ (Table S2). Meanwhile, CF@g-C_3_N_4_–Ru NPs exhibits a decreased surface area and pore volume due to the blocking of TiO_2_. The mesoporosity, defined as V_meso_/V_p_, is determined to be 60.0%, which is lower than that for CF@g-C_3_N_4_–Ru SAs (71.9%). This may coincide with the removal of partial g-C_3_N_4_ upon the secondary pyrolysis that slightly erodes the hierarchical structure. On the other hand, CF–Ru is mesoporous, and gives an extremely high surface area of 587 m^2^ g^−1^, probably due to the removal of TiO_2_, which acts as the template for micro-mesopores, while g-C_3_N_4_–Ru is determined to be microporous, showing an extremely low surface area of 16 m^2^ g^−1^ and a low pore volume of 0.09 cm^3^ g^−1^. These results demonstrate that CF is dominating in contributing to the porosity of CF@g-C_3_N_4_–Ru SAs.

**Figure 4:**
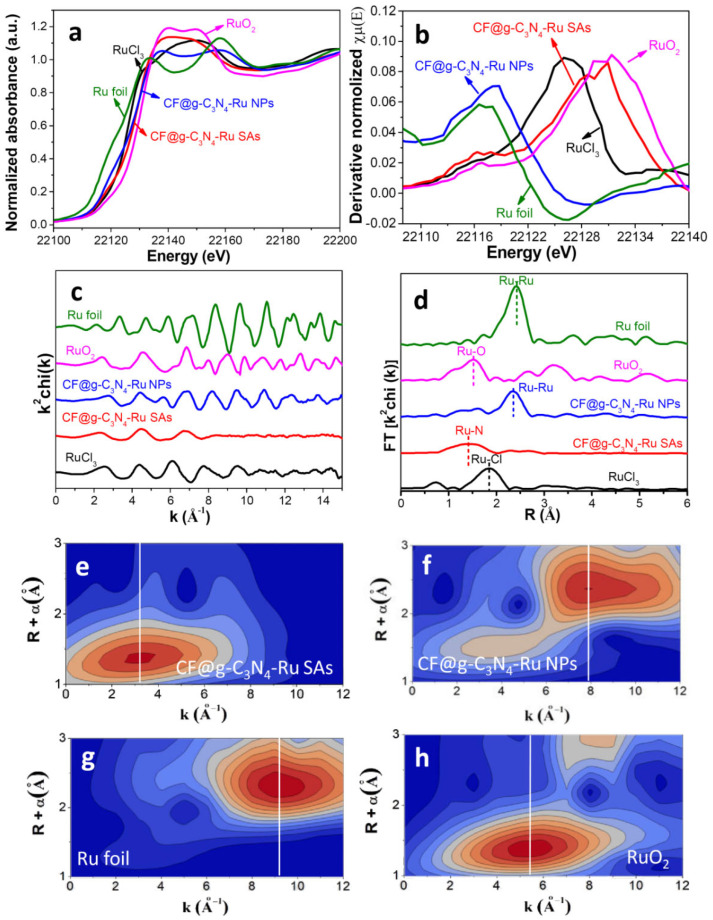
(**a**) Ru K-edge XANES, (**b**) the first derivative curves, (**c**) k^2^-weighted EXAFS spectra at k space, (**d**) the corresponding Fourier transformed EXAFS spectra, and (**e**–**h**) WT for the k^2^-weighted EXAFS signal of different Ru-containing samples.

In order to investigate the electronic structure and coordination configuration of the Ru species, an element-specific XAFS analysis was performed. As shown in Figure 4a, the absorption edge position of CF@g-C_3_N_4_–Ru SAs is between that of Ru and RuO_2_, suggesting that the Ru single atoms carry some positive charges [23]. These Ru single atoms may interact with the CF@g-C_3_N_4_ support by donating electrons to the surrounding N moieties. The derivative profile for CF@g-C_3_N_4_–Ru SAs exhibits the edge structure ranging from 22,126 to 22,131 eV. Additionally, the maximum slope is located at 22,130 eV, between that of RuCl_3_ and RuO_2_ (Figure 4b). Therefore, the valence state of the Ru species in CF@g-C_3_N_4_–Ru SAs is +3–+4 [12]. On the contrary, the absorption edge and derivative profile for CF@g-C_3_N_4_–Ru NPs match well with those for Ru foil, demonstrating the formation of the metallic Ru species. In the corresponding k space, the *k*^2^*χ*(*k*) oscillation curve of CF@g-C_3_N_4_–Ru SAs shows a reduction in the oscillation amplitude at a higher *k* region (*k* > 8 Å^−^^1^) compared to that of Ru foil (Figure 4c), indicating the dominance of low Z backscatters, which would be N/C in our system [24]. As for CF@g-C_3_N_4_–Ru NPs, the strong oscillations at a high k region (k > 8 Å^−^^1^) confirm the predominant Ru–Ru pairs (Figure 4c). Figure 4d shows the Fourier transformed (FT) *k*^2^-weighted extended X-ray absorption fine structure (EXAFS) spectra of CF@g-C_3_N_4_–Ru SAs and the reference Ru foil, RuO_2_ and RuCl_3_. There is only one prominent peak at 1.4 Å, which can be assigned to the Ru–N contribution [15], adjacent to the Ru–O scattering path at 1.5 Å as observed for RuO_2_ [8]. No peaks can be observed at ~2.6 and 1.8 Å corresponding to Ru–Ru and Ru–Cl pairs as shown for Ru foil and RuCl_3_, respectively [25]. This suggests the pristine Ru–Cl pair was broken to construct Ru–N coordination, forming atomically dispersed Ru species. Comparatively, the radical distribution of CF@g-C_3_N_4_–Ru NPs obtains a dominated Ru–Ru pair (2.6 Å) combined with Ru–N contribution (1.5 Å), implying that the metallic Ru is stabilized by the surrounding N moieties. In order to further explore the dispersion of the Ru species in CF@g-C_3_N_4_–Ru SAs, we performed wavelet transform (WT) of K-edge EXAFS. In Figure 2e, CF@g-C_3_N_4_–Ru SAs shows only one intensity maximum at 4.0 Å^−1^, ascribed to the Ru–N coordination. Additionally, no intensity maximum related to the Ru–Ru and Ru–O contributions can be found as compared to the WT plots of CF@g-C_3_N_4_–Ru NPs, Ru foil and RuO_2_ (Figure 2f–h). In order to further obtain the structure parameter of the Ru species, we conducted a least-squares EXAFS fitting. As illustrated in Figure S2 and Table 1, the coordination number of Ru–N for CF@g-C_3_N_4_–Ru SAs is approximately 5.3, and the mean bond length of Ru–N is 2.03 Å. This suggests that the Ru–N bonding in CF@g-C_3_N_4_–Ru SAs may adopt a square pyramidal configuration [26]. A coordination number for the Ru–N is 1.4 and the second shell Ru–Ru is 8.8 for CF@g-C_3_N_4_–Ru NPs, indicating that metallic Ru species are partially stabilized by N-coordination.

**Figure 5:**
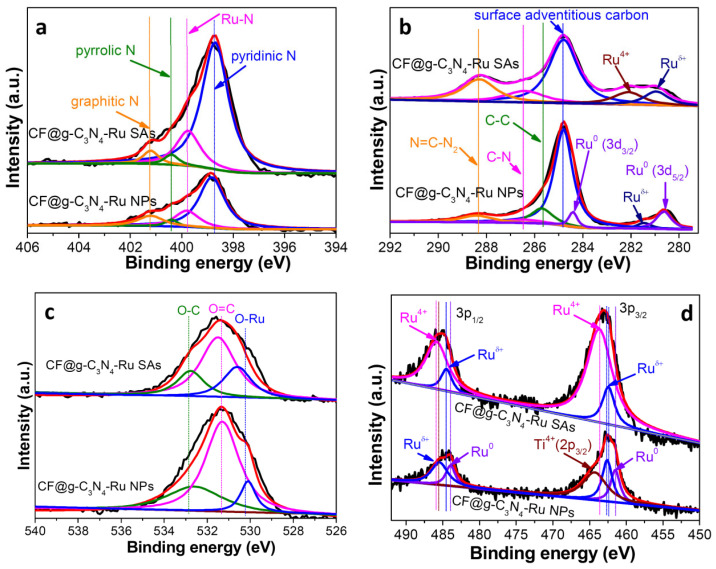
High-resolution (**a**) N 1s, (**b**) C 1s, (**c**) O 1s and (**d**) Ru 3p spectra for CF@g-C_3_N_4_–Ru SAs and CF@g-C_3_N_4_–Ru NPs.

The surface composition and chemical state of different samples are investigated by X-ray photoelectron spectroscopy (XPS). The C, O, N and Ru elements can be found for both CF@g-C_3_N_4_–Ru SAs and CF@g-C_3_N_4_–Ru NPs (Figure S3). The high-resolution N 1s spectrum of CF@g-C_3_N_4_–Ru SAs can be deconvoluted into four peaks with binding energies of 398.8, 399.7, 400.4 and 401.2 eV (Figure 5a), corresponding to pyridinic-N, Ru–N, pyrrolic-N and graphitic-N, respectively [27]. Interestingly, the Ru–N signal for CF@g-C_3_N_4_–Ru SAs is negatively shifted by 0.1 eV compared to that for CF@g-C_3_N_4_–Ru NPs. This agrees with the positive Ru atoms that can denote more electrons to the surrounding N moieties, while the metallic Ru species shows a weak electron-denoting capability. The N moieties can be further confirmed by the asymmetric signals of C-N at 286.5 eV and N=C-N_2_ at 288.3 eV in the C 1s spectrum [28], as shown in Figure 5b. Three oxygen functionalities including O–C, O–H and Ru–O were found at the binding energies of ca. 532.8, 531.5 and 530.6 eV [29], respectively, as shown in Figure 5c. These results demonstrate that the carbon skeleton is decorated with some oxygen functionalities. Owing to the overlapping of C 1s and Ru 3d electrons, the binding energies at 280.9 and 282.1 eV can be ascribed to 3d_5__/2_ of Ru^δ+^ (0 < δ < 4) and Ru^4^^+^ for CF@g-C_3_N_4_–Ru SAs, while those at 280.6, 281.4 and 284.4 eV correspond to 3d_5/2_ of Ru^0^ and Ru^δ+^, and 3d_3/2_ of Ru^0^ for CF@g-C_3_N_4_–Ru NPs [30,31]. Since the binding energy of Ru 3d_3/2_ (ca. 284.4 eV) is too close to C 1s (284.6 eV) to be distinguished, the electronic state of the surface Ru species was determined by the binding energy of Ru 3p. For CF@g-C_3_N_4_–Ru SAs, the binding energies at 463.7 and 485.9 eV correspond to Ru^4+^ (83%) of Ru 3p_3/2_ and Ru 3p_1/2_, while those at 462.4 and 484.5 eV can be ascribed to the positively charged Ru^δ+^ (17%, 0 < δ < 4) [11]. Comparatively, the deconvoluted Ru 3p spectrum of CF@g-C_3_N_4_–Ru NPs exhibits binding energies at 461.8 and 483.8 eV for Ru^0^ (15%) and those at 462.6 and 484.6 eV correspond to Ru^δ+^ (25%). An additional peak at 464.3 eV is present, due to the overlapping of Ru 3p and Ti 2p, which can be ascribed to 2p_3/2_ of Ti^4+^ [32]. These results demonstrate that the atomically dispersed Ru atoms are mainly surrounded by N moieties and are thus positively charged, showing a higher valence, while the Ru NPs possess both metallic and N-coordinated Ru species. Furthermore, the Ru content is determined to be 9.7 wt% by XPS analysis, which is much larger than that estimated by ICP-OES (Table S1), implying that the Ru species are mainly located outside, confirming the TEM observation.

The hydrogenation of LA was performed under bath reactions in water to investigate the catalytic property of various Ru-containing catalysts. As listed in Table 2, similar to the blank reaction (entry 1), LA conversion can be ignored as CF@g-C_3_N_4_ was involved (entry 2), since no active Ru species are involved. Figure S4 shows the time-on-line concentration profiles for LA. As shown, all Ru-containing catalysts are active for LA hydrogenation, suggesting the importance of Ru species in LA hydrogenation reactions. Additionally, the LA conversion increased as the reaction time was prolonged, and then was nearly completed as the reaction time was extended to 1.3 h. GVL was found to be the main product over all Ru-containing catalysts, and trace 1,4-pentanediol (PD) and 2-methyltetrahydrofuran (MTHF) were also detected as consecutive hydrogenation reaction products. CF@g-C_3_N_4_–Ru NPs displays a higher catalytic activity under identical reaction conditions, delivering an LA conversion of 100% and a GVL yield of 99% for the duration of 2 h. This corresponds to a TOF value of 0.180 s^−1^, which is higher than 0.062 s^−1^ as CF@g-C_3_N_4_–Ru SAs was utilized. As previously reported, the TOF value as the single-atom Ru catalyst involved (0.17 s^−1^) is also lower than that for the nanoparticle catalyst (0.82 s^−1^) at 413 K and 10 bar H_2_ [14]. As demonstrated, H_2_ dissociation is a prerequisite for hydrogenation reactions, which is more important than C=O group activation. However, the H_2_ dissociation over single atoms (like Pd) was significantly retarded compared to that over nanoparticles [33]. The lower catalytic activity for CF@g-C_3_N_4_–Ru SAs as compared to that for the Ru nanoparticle catalyst in this study agrees with the results in the literature [34,35]. Nevertheless, CF@g-C_3_N_4_–Ru SAs delivers a GVL yield of 99%, which is higher than that detected for g-C_3_N_4_–Ru (88%) and CF–Ru (92%), as LA was completely converted and some side products such as PD and MTHF were produced. The superior selectivity of GVL achieved by CF@g-C_3_N_4_–Ru SAs may correlate with the one-dimensional hierarchical supporting matrix, which provides well-defined pore arrays, facilitating the directional synthesis of GVL. This can also be confirmed by the high selectivity of 99%, as the nanoparticle catalyst CF@g-C_3_N_4_–Ru NPs was involved. A reaction scheme can be proposed as follows: when interacted with Ru single atoms, the active H^*^ is provided by the dissociation of molecular hydrogen (H_2_). The C=O group in LA and H^*^ are separately adsorbed on N and Ru centers, and activated to form 4-hydroxypentanoic acid (4-HPA) as an intermediate product. The produced 4-HPA is unstable and can be easily converted into GVL via intramolecular lactonization, especially at a relatively high temperature or in acidic/basic reaction media.

**Figure 6:**
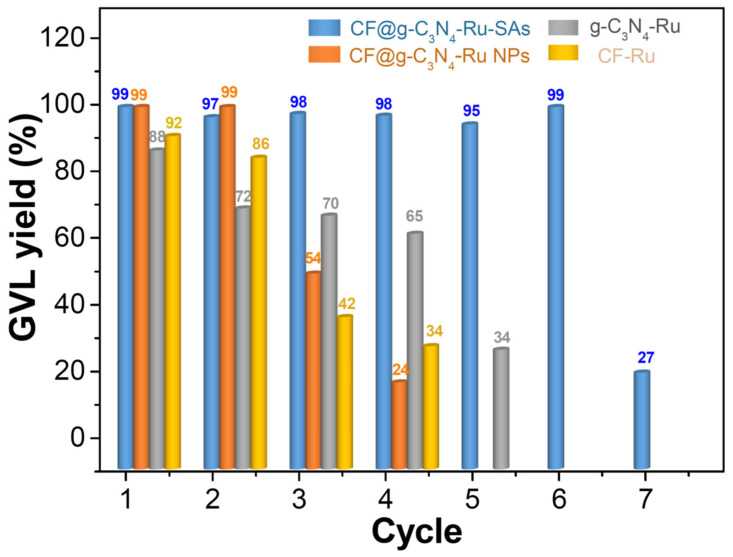
Cycling tests for different Ru-containing catalysts.

The cycling stability of metal catalysts is important for their potential industry applications. To examine the stability of CF@g-C_3_N_4_–Ru SAs, cycling experiments were also carried out for other Ru-containing catalysts for comparison. As illustrated in Figure 6, CF@g-C_3_N_4_–Ru SAs was quite stable and could be used at least six times without a significant loss of GVL yield in water. The Ru concentration in the filtrate is 0.004 ppm estimated by ICP-OES (Table S1). The TEM observation for the spent CF@g-C_3_N_4_–Ru SAs demonstrates that the aggregation to Ru nanoparticles did not occur, and the hierarchical skeleton was almost intact after seven runs (Figure S5). These results are indicative of the highly stable CF@g-C_3_N_4_–Ru SAs toward the aqueous LA hydrogenation in acidic media (pH ≈ 1). For comparison, CF@g-C_3_N_4_–Ru NPs can be cycled twice and delivers a GVL yield of 99%, which is decreased by 35% at the third run. Additionally, the content of leached Ru is four times that of CF@g-C_3_N_4_–Ru SAs, as estimated by ICP-OES. Such a severe exfoliation is in accordance with the observation of excess leached Ru NPs as ultrasonic treatment is employed for CF@g-C_3_N_4_–Ru NPs. TEM observation for the spent CF@g-C_3_N_4_–Ru NPs reveals the aggregation of Ru NPs, though their hierarchical skeleton is almost intact (Figure S5). N_2_ adsorption reveals that both spent CF@g-C_3_N_4_–Ru SAs and CF@g-C_3_N_4_–Ru NPs show a decreased mesopore size of 2.4 and 2.1 nm, respectively (Figure S6). Additionally, the microporous structure in fresh catalysts disappears upon cycling. Meanwhile, the specific surface area is decreased by 29.7 and 26.7% for spent CF@g-C_3_N_4_–Ru SAs and CF@g-C_3_N_4_–Ru NPs, respectively (Table S1). Therefore, the shielding effect of reagents retained after the reaction should be responsible for the degradation of the porous structure. More Ru^δ+^ species (84.8%) are detected for spent CF@g-C_3_N_4_–Ru SAs, which is different from the dominated Ru^4+^ in the fresh catalyst (83.0%) (Figure S7). This implies that in the presence of H_2_, most Ru^4+^ species evolve into Ru^δ+^ after cycling. As for spent CF@g-C_3_N_4_–Ru NPs, the content of Ru^δ+^ (43.3%) is similar to that of the fresh catalyst, while the atomic ratio of Ru^0^ is increased by 14.5% (Figure S7). This leads to the rapid decrease in GVL yield, and confirms that Ru^δ+^ is the actual active site and is dominated during the hydrogenation of LA. Based on these investigations, it can be inferred that the successive reduction in the Ru^δ+^ species can be mainly responsible for the high stability of CF@g-C_3_N_4_–Ru SAs. On the other hand, it is also found that CF–Ru can be cycled twice, and only 54% of GVL yield is obtained at the third run. Owing to the absence of N-coordination, the anchored Ru species are easily leached, leading to a rapid decrease in activity. On the contrary, the g-C_3_N_4_–Ru catalyst exhibits a stepwise deactivation during cycling, with a drop in LA conversion from 100 to 70% as well as in GVL yield from 88 to 66% after four runs (Figure 6). A sharp decrease in GVL yield (32%) was observed for g-C_3_N_4_–Ru at the fifth run. Varma and his coworkers have reported a similar deactivation for the AgPd@g-C_3_N_4_ catalyst, which displays a steady decline in GVL yield from 42 to 34% after four cycles at 473 K in H_2_ atmosphere (35 bar) [36]. The strong interaction between metal and g-C_3_N_4_ may slow down the deactivation. However, it is noteworthy that g-C_3_N_4_–Ru is less stable than CF@g-C_3_N_4_–Ru SAs in the aqueous and acidic media, suggesting that the hierarchical pore arrays can facilitate the mass transfer and thus retard the active site poisoning in the presence of impurities produced during reactions. Therefore, the unprecedented stability of CF@g-C_3_N_4_–Ru SAs can be ascribed to the following synergism: (1) The construction of N-isolated Ru atoms retards the Ru sintering, leaching and acid poisoning under harsh reaction conditions, and an over oxidation/reduction when exposed to air/H_2_ is avoided and, thus active Ru^δ+^ species are continuously created during reactions to retain a high activity. (2) The one-dimensional hierarchy CF@g-C_3_N_4_ combines the merits of mesoporous carbon and graphitic carbon nitride. Mesopores provide superior diffusion paths for LA/GVL diffusion, while N coordination sites not only stabilize Ru atoms, but also promote LA adsorption and thus promote its contact with active sites together with mesoporous carbon. Furthermore, the interaction with CF improves the dispersion of g-C_3_N_4_, which increases the Ru atom accessibility during reactions. The synergism between dynamically stable Ru atoms and a firm one-dimensional hierarchy thereby improves the overall catalytic stability of CF@g-C_3_N_4_–Ru SAs under harsh reaction conditions.

## 4. Conclusions

In summary, we have successfully developed a stable LA hydrogenation catalyst with Ru single atoms anchored on a one-dimensional CF@g-C_3_N_4_ hierarchy. A deep investigation reveals that the durability of the obtained CF@g-C_3_N_4_–Ru SAs catalyst highly depends on single Ru atoms and the core–shell structured hierarchy, as well as their ensemble effect, verified by various characterization techniques and comparative catalytic tests. The elaborately fabricated CF@g-C_3_N_4_–Ru SAs catalyst demonstrates unprecedented catalytic stability for aqueous LA hydrogenation in acidic media. This is probably due to the fact that the N-coordinated Ru sites are isolated and are free from acid poisoning, and the hierarchical pore arrays facilitate the mass transfer and thereby retard the deactivation of active sites. The development of such an efficient CF@g-C_3_N_4_–Ru SAs catalyst successfully proves the concept of metal- and support- dependent durability, and also gives a strong indication of its generality in the fabrication of a variety of stable single-atom catalysts.

## Figures and Tables

**Table 1:** Fitting parameters for Ru-containing samples.

Sample	Absorber- Scatter Pair	Coordination Number	Bond Distance (Å)	[σ^2^] ^a^	ΔE_0_^a^ (eV)	R Factor
CF@g-C_3_N_4_–Ru SAs	Ru-N	5.3	2.03	0.0081	0.600	0.023
CF@g-C_3_N_4_–Ru NPs	Ru-N	1.4	1.95	0.0007	−8.50	0.019
	Ru-Ru	8.8	2.68	0.0055	−3.70	
RuO_2_	Ru-O	4.0	1.98	0.0014	0.279	0.029
	Ru-Ru	2.0	3.10	0.0030	−8.075	
RuCl_3_	Ru-Cl	6.0	2.36	0.0075	−0.873	0.099
	Ru-Ru	2.0	2.98	0.0130	−9.249	
Ru foil	Ru-Ru	12.0	2.68	0.0048	3.663	0.007

^a^ σ^2^ = Debye-Waller factor and ΔE_0_ = inner potential correction.

**Table 2:** Summary of the catalytic performance for different catalysts.

Entry	Catalyst	LA Conversion ^a^ (%)	Selectivity to GVL (%)	Yield of GVL (%)	TOF (s^−1^) ^c^
1	Blank	2	- ^b^	-	-
2	CF@g-C_3_N_4_	5	-	-	-
3	CF@g-C_3_N_4_–Ru NPs	100	99	99	0.180
4	CF@g-C_3_N_4_–Ru SAs	100	99	99	0.062
5	g-C_3_N_4_-Ru	100	88	88	0.025
6	CF-Ru	100	92	92	0.040

^a^ Reaction conditions: LA 0.12 g (1 mmol), catalyst 30 mg, H_2_ 4.5 MPa, water 40 mL, 2 h and 150 °C. ^b^ Not detected. ^c^ TOF = mol_LA_/(mol_Ru_ × h) at a LA conversion of 10%.

## Data Availability

Not applicable.

## Supplementary Materials

The following supporting information can be downloaded at: https://www.mdpi.com/article/10.3390/ma15217464/s1, Table S1. The atom percentage and elemental analysis for different Ru-containing catalysts, Table S2. Textural properties of various Ru-containing catalysts, Figure S1. TEM images for (a) CF@g-C3N4–Ru NPs and (b) filtrate obtained by ultrasonic treatment of CF@g-C3N4–Ru NPs in ethanol, with the corresponding particle size distribution histogram (inset), Figure S2. Curve fitting for (a) CF@g-C3N4–Ru SAs, (b) CF@g-C3N4–Ru NPs, (c) RuCl3, (d) RuO2 and (e) Ru foil, Figure S3. XPS survey scan for (a) CF@g-C3N4–Ru SAs and (b) CF@g-C3N4–Ru NPs, Figure S4. Time profiles for catalytic conversion of LA in water using different Ru-containing catalysts, Figure S5. TEM images of (a,b) spent CF@g-C3N4–Ru SAs and (c,d) spent CF@g-C3N4–Ru NPs, Figure S6. N2 adsorption/desorption isotherm and the corresponding pore size distribution curve (inset) for (a) spent CF@g-C3N4–Ru SAs and (b) CF@g-C3N4–Ru NPs, Figure S7. High-resolution Ru 3p spectra for (a) spent CF@g-C3N4–Ru SAs and (b) spent CF@g-C3N4–Ru NPs.
