# Supplementary material for: Ru Single Atoms on One-Dimensional CF@g-C3N4 Hierarchy as Highly Stable Catalysts for Aqueous Levulinic Acid Hydrogenation
Source: Materials (Basel). 2022 Oct 25;15(21):7464. doi: 10.3390/ma15217464 (PMC9658288; doi:10.3390/ma15217464)
Supplement: Supplementary file 1 [file materials-15-07464-s001.zip › materials-1961079-supplementary.pdf]

Supporting Information

Ru Single Atoms on One-Dimensional CF@g-C_3N_4 Hierarchy as Highly Stable Catalysts for Aqueous Levulinic Acid Hydrogenation

Ying Yang [1,*], Suoying Zhang [2], Lin Gu [1] and Shijie Hao [1,*]

[1] State Key Laboratory of Heavy Oil Processing, China University of Petroleum, Changping,
Beijing 102249, China
[2] Institution of Advanced Materials, Nanjing Tech University, Nanjing 211816, China
* Correspondence: yyang@cup.edu.cn (Y.Y.); haoshijie@cup.edu.cn (S.H.); Tel.: +86-10-89734979 (Y.Y.)

Table S1 The atom percentage and elemental analysis for different Ru-containing catalysts

Catalyst	Percentage of different elements[a] (at. %)				Ru loading[b] (wt%)	Ru loss[b] (ppm)
	C	N	O	Ru		
CF@g-C_3N_4-Ru SAs	55	18	20	7	3.0	0.004
CF@g-C_3N_4-Ru NPs	51	28	10	11	1.3	0.016
g-C_3N_4-Ru	-	-	-	-	4.5	-
CF-Ru	-	-	-	-	4.1	-

[a]Estimated by XPS.

[b]Detected by ICP-OES analysis.

Table S2 Textural properties of various Ru-containing catalysts

Catalyst	S_{BET} [a] (m^2 g^{-1})	V_p [b] (cm^3 g^{-1})	d_{meso} [c] (nm)	V_{micro} [d] (cm^3 g^{-1})	d_{micro} [e] (nm)	$X_{mesoporosity}$ [f] (%)
CF@g-C_3N_4-Ru SAs	380	0.57	3.7	0.161	0.52	71.9
CF@g-C_3N_4-Ru NPs	266	0.25	3.5	0.103	0.60,0.65	60.0
g-C_3N_4-Ru	16	0.09	-	0.007	-	-
CF-Ru	587	0.30	3.4	0.227	0.51,0.62	23.3
spent CF@g-C_3N_4-Ru SAs	267	0.219	2.4	-	-	
spent CF@g-C_3N_4-Ru NPs	195	0.159	2.1	-	-	

[a]The BET surface area was obtained from the adsorption branches in the relative pressure range of 0.05~0.29.

[b]The single point adsorption total pore volume was taken at the relative pressure of 0.99.

[c]The mesopore size distribution was calculated from the desorption branches by the Barret-Joyner-Halenda (BJH) method.

[d]The single point adsorption micropore volume was taken at the diameter less than 2 nm.

[e]The micropore size distribution was estimated by the Horvath-Kawazoe (H-K) method.

[f]The mesoporosity is defined as the ratio of V_{meso}/V_p, and was calculated according to the equation, $X_{mesoporosity} = (1-V_{micro}/V_p) \times 100\%$.

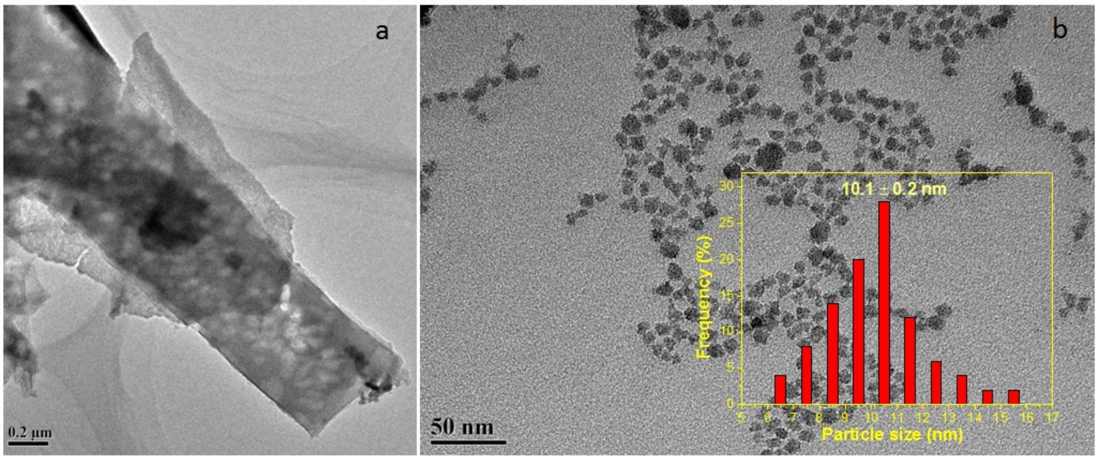

Figure S1. TEM images for (a) CF@g-C_3N_4-Ru NPs and (b) filtrate obtained by ultrasonic treatment of CF@g-C_3N_4-Ru NPs in ethanol, with the corresponding particle size distribution histogram (inset).

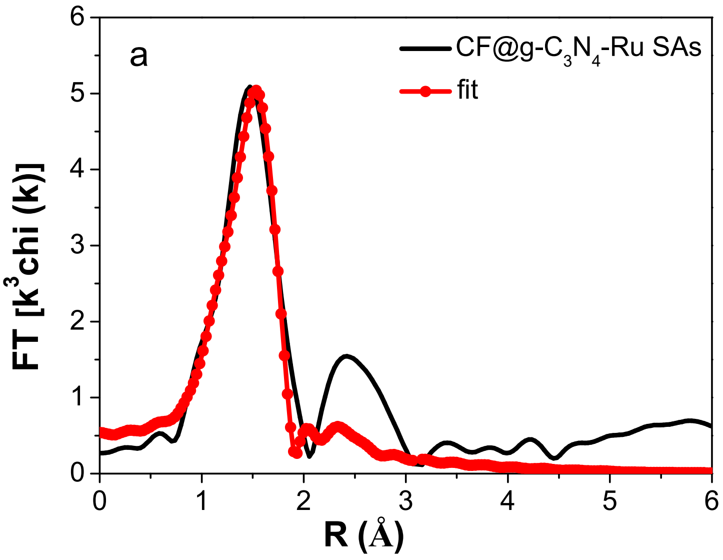

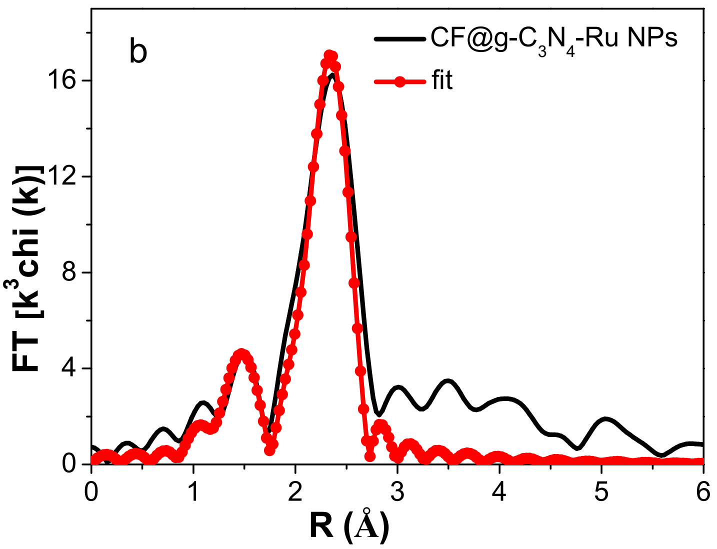
b
CF@g-C3N4-Ru NPs
fit
FT [k3chi (k)]
R (Å)
0
4
8
12
16
0
1
2
3
4
5
6

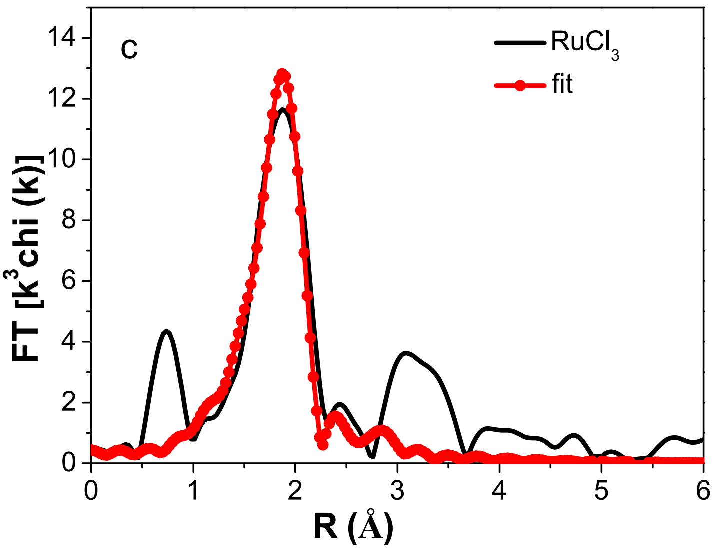
c
RuCl3
fit
FT [k3chi (k)]
R (Å)
0
2
4
6
8
10
12
14
0
1
2
3
4
5
6

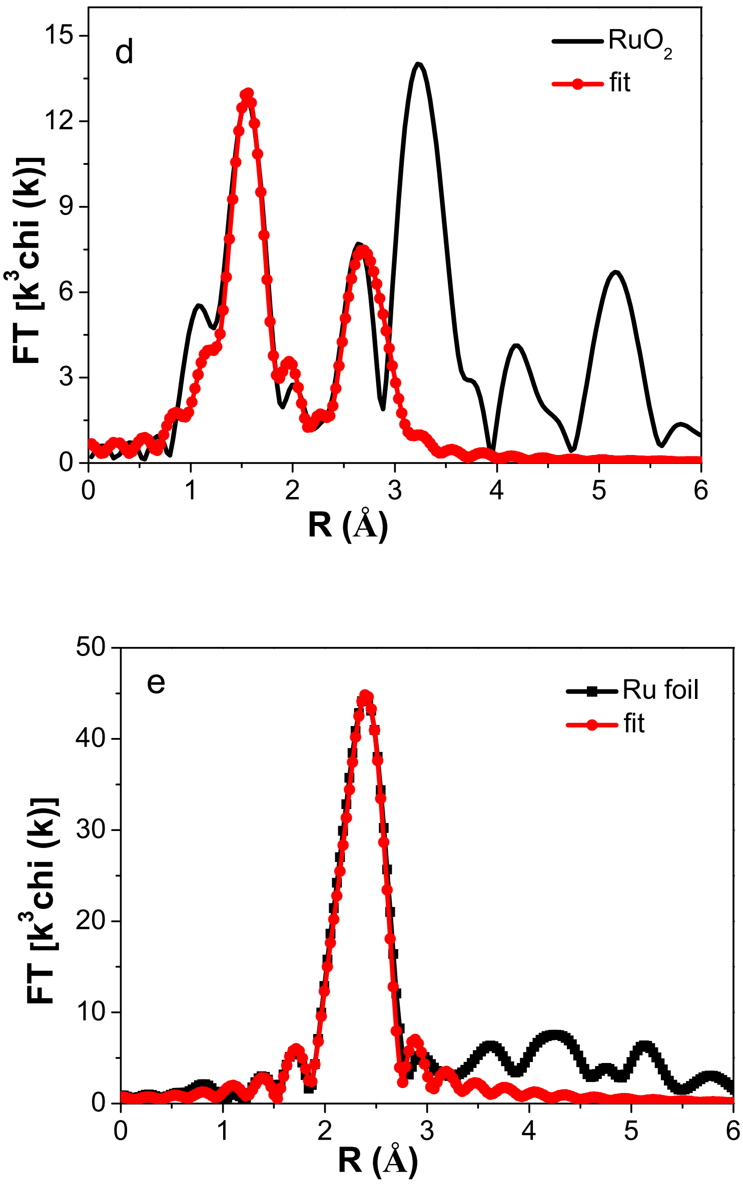

Figure S2. Curve fitting for (a) CF@g-C_3N_4-Ru SAs, (b) CF@g-C_3N_4-Ru NPs, (c) $RuCl_3$, (d) RuO_2 and (e) Ru foil.

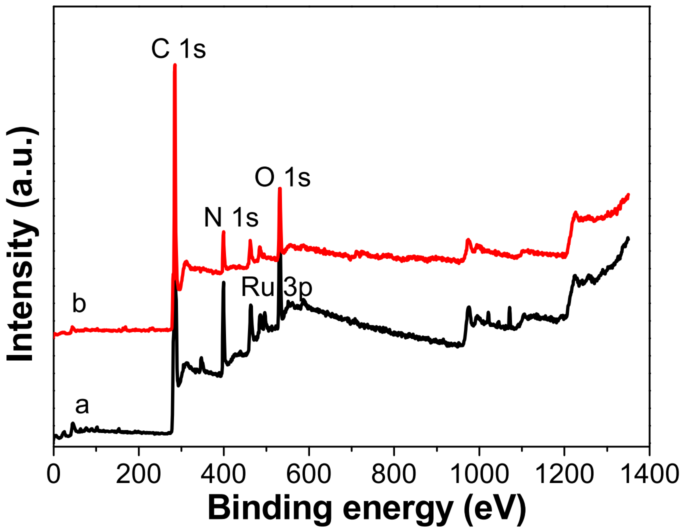

Figure S3. XPS survey scan for (a) CF@g-C_3N_4-Ru SAs and (b) CF@g-C_3N_4-Ru NPs.

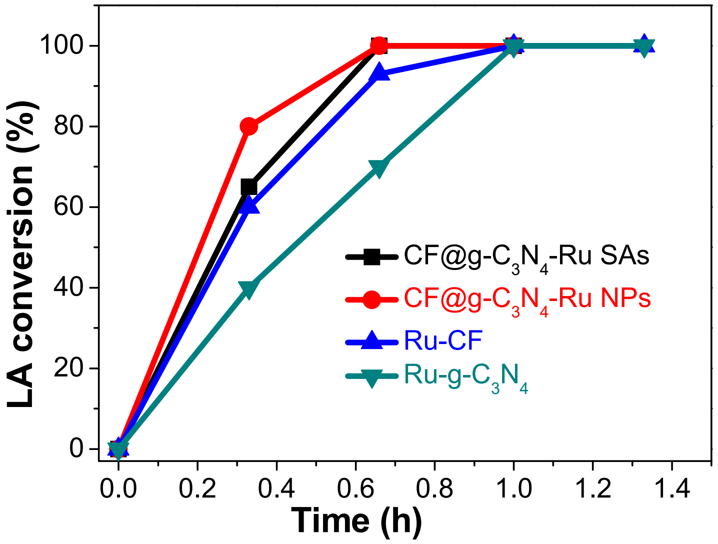

Figure S4. Time profiles for catalytic conversion of LA in water using different Ru-containing catalysts.

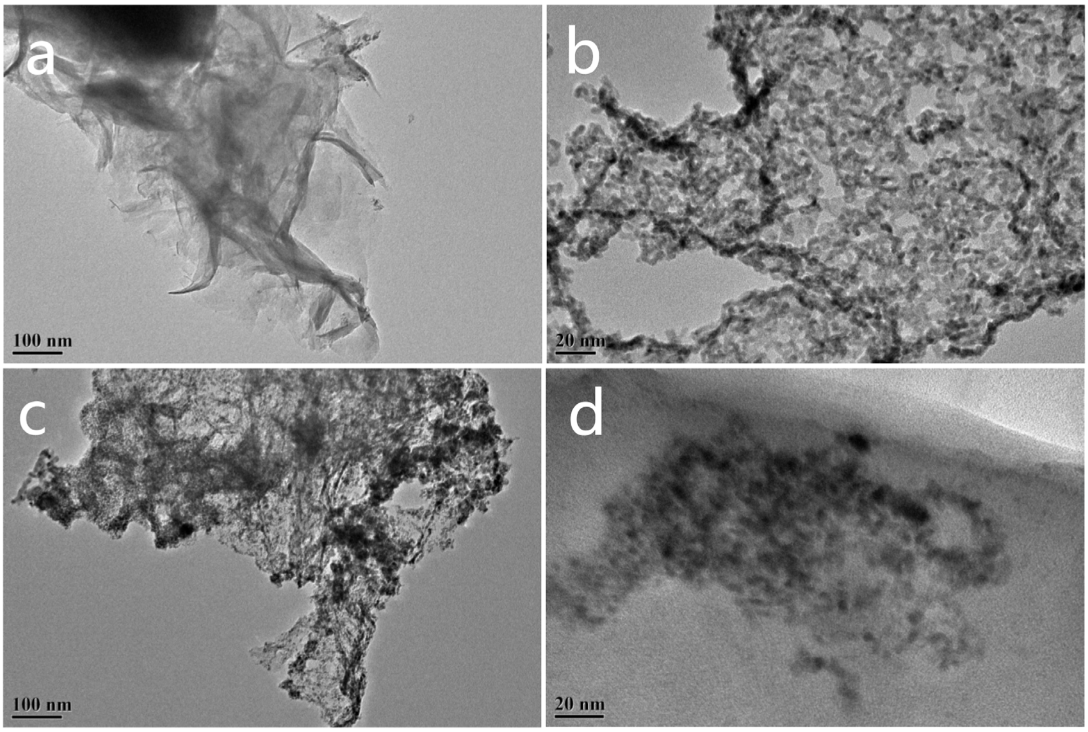

Figure S5. TEM images of (a,b) spent CF@g-C_3N_4-Ru SAs and (c,d) spent CF@g-C_3N_4-Ru NPs.

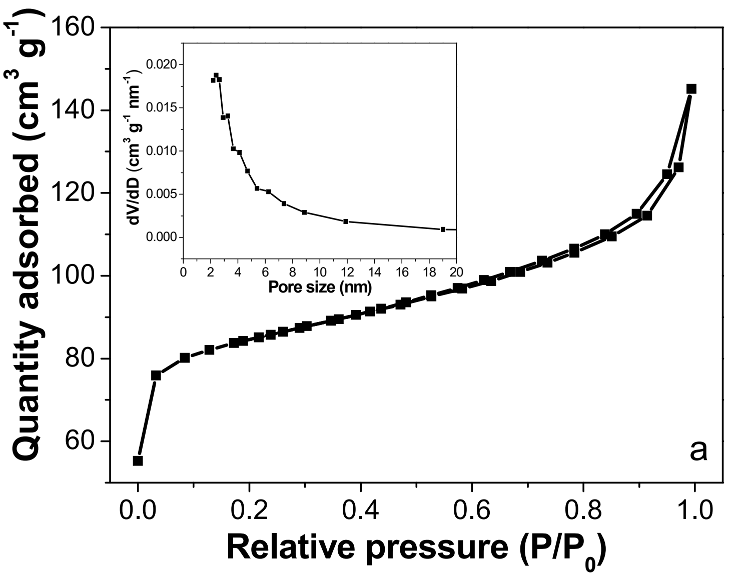

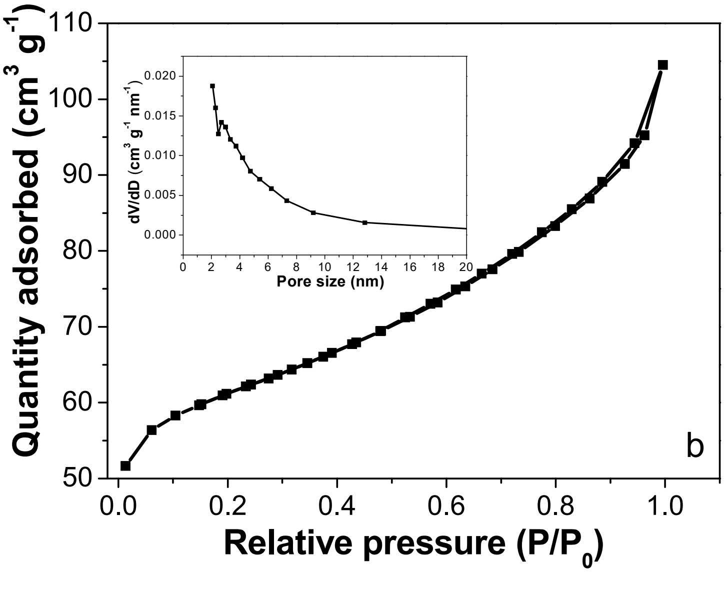

Figure S6. N_2 adsorption/desorption isotherm and the corresponding pore size distribution curve (inset) for (a) spent CF@g-C_3N_4-Ru SAs and (b) CF@g-C_3N_4-Ru NPs.

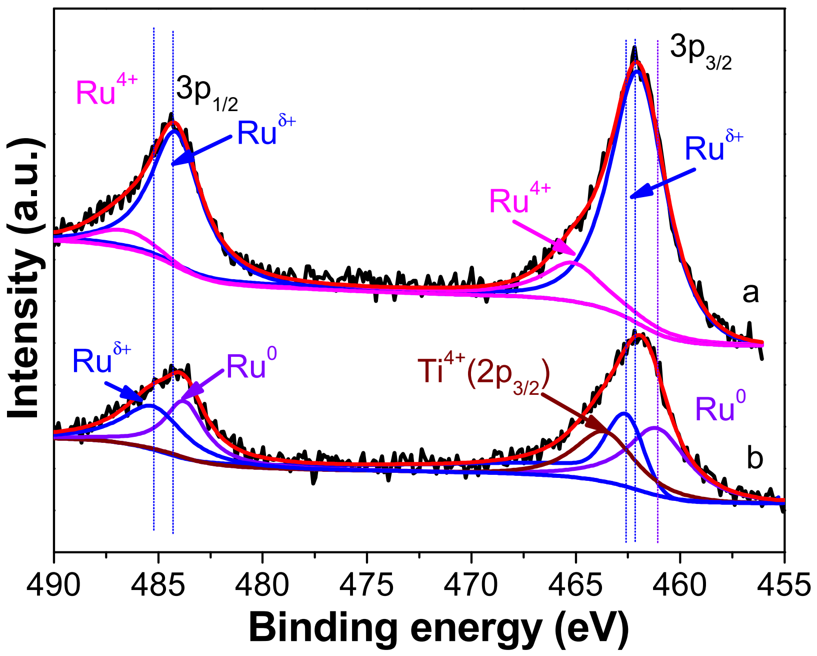

Figure S7. High-resolution Ru 3p spectra for (a) spent CF@g-C_3N_4-Ru SAs and (b) spent CF@g-C_3N_4-Ru NPs.
